# Evidence of the efficacy and safety of house dust mite subcutaneous immunotherapy in elderly allergic rhinitis patients: a randomized, double-blind placebo-controlled trial

**DOI:** 10.1186/s13601-017-0180-9

**Published:** 2017-12-01

**Authors:** Andrzej Bożek, Krzysztof Kołodziejczyk, Renata Kozłowska, Giorgio Walter Canonica

**Affiliations:** 1 Clinical Department of Internal Disease, Dermatology and Allergology in Zabrze, Medical University of Silesia, MC Sklodowskiej 10, 41-800 Zabrze, Katowice, Poland; 2 Allergic Diseases Monitoring Association AMAD, Mikolow, Poland; 3 Allergy Outpatient Clinic Katowice, Katowice, Poland; 4 Humanitas University, Milan, Italy

**Keywords:** Immunotherapy, Allergic rhinitis, Elderly, House dust mite

## Abstract

**Background:**

Allergen specific immunotherapy (AIT) in elderly patients is controversial, and there is still little evidence supporting the safety and efficacy of this treatment in this population. The study objective was to evaluate the safety and efficacy of AIT for house dust mite allergens in patients over 65 years of age with allergic rhinitis (AR) and a documented allergy to house dust mites. The primary endpoint was the change from baseline in the mean average adjusted symptom score (AAdSS) and the total combined rhinitis score (TCRS) difference in the least square means for the label compared to placebo.

**Methods:**

Fifty-eight AR elderly patients who were monosensitized to house dust mites were individually randomized in comparable numbers to one of two parallel groups with the following interventions: 2 years of perennial AIT using PURETHAL Mites or placebo. The symptoms and medication scores were presented as the AAdSS and TCRS. Quality of life, based on the rhinoconjunctivitis quality of life questionnaire (RQLQ), nasal allergen provocation responsiveness, serum allergen-specific IgG4 to *D. pteronyssinus* and *D. farinae* and *Der p1* and *Der p2* were monitored. The intent-to-treat population was analysed.

**Results:**

After 24 months of AIT, AAdSS significantly decreased from 4.27 ± 1.58 to 1.82 ± 0.71 (*p* < 0.05). The TCRS was significantly decreased after 2 years of AIT. Serum-specific IgG4 against *D. pteronyssinus*, *D. farinae, Der p1*, and *Der p2* increased during the AIT trial in the study group. The RQLQ score was significantly improved in patients who received AIT, from 1.86 (95% CI 1.51–1.78) to 1.26 (95% CI 1.09–1.55). Two mild systemic anaphylactic reactions (degree I) were reported after injections in the active group during the AIT therapy.

**Conclusion:**

The DBPC trial showed AIT for house dust mite allergens was effective and safe in elderly patients with allergic rhinitis.

**Trial registration:**

This randomized, double-blinded placebo-controlled (DBPC) trial was conducted at one centre (ClinicalTrials.gov no. NCT03209245)

## Background

Allergic rhinitis is often underestimated in elderly patients. The guidelines addressing the diagnosis and treatment of allergic diseases rarely focus on the elderly population and often ignore this population completely. However, allergic rhinitis and asthma caused by inhaled allergens, such as house dust mites (HDMs), are more common in patients over 65 years of age [1, 2]. Allergen-specific immunotherapy (AIT) has provided a safe and effective treatment method, particularly for allergic rhinitis. Several studies have confirmed the efficacy of this therapy in young people [3, 4]. HDMs are a major allergen for patients with allergies, and several randomized controlled immunotherapy trials exhibiting a high degree of safety and efficacy have been reported [5–8]. Despite the lack of objective contraindications, specific immunotherapy has not played a significant role in elderly patients, which may be due to the lack of safety evidence in this group. Few studies have confirmed that AIT, primarily sublingual, is safe and effective in elderly patients [9, 10]. Finally, there is also a question of whether immunotherapy can produce sufficient allergen tolerance in patients with aged immune responses.

The objective of this study was to assess the safety and efficacy of subcutaneous HDM allergens in elderly patients with allergic rhinitis due to HDM.

## Methods

### Study design

This study was a randomized, double-blind, placebo-controlled (parallel—group trial) conducted at one centre. The study was approved by the local ethics committees of the Medical University of Silesia in Poland. All patients provided signed informed consent. The trial was registered on ClinicalTrials.gov under Protocol Record NCT03209245.

### Participants

Participants were enrolled between May and July 2014. First, there was a need for pre-screening of approximately 157 patients with inhalant allergies and who were the right age. The following eligibility criteria were applied:

- patients with moderate or severe intermittent allergic rhinitis and who fulfilled the allergic rhinitis and its impact on asthma (ARIA) criteria [11],
- a positive skin prick test (SPT) and a positive result of specific immunoglobulin E (sIgE) to *D. pteronyssinus* and *D. farinae* allergens, and
- a nasal provocation test (NPT) positive for *D. pteronyssinus* and *D. farinae* allergens. The exclusion criteria were a clinical allergy and/or a positive skin prick test and specific IgE to other inhalant allergens, diagnosis of bronchial asthma, non-allergic rhinitis and severe non-stable diseases, other nasal problems, such as chronic nasal obstruction, reduced olfaction, bacterial colonization, and chronic sinusitis, and other chronic or acute clinical disorders or a history of respiratory tract infections within 4 weeks of the study. However, patients with stable coronary disease, diabetes, and arterial hypertension were permitted in the study. All subjects were required to abstain from anti-allergy drugs and glucocorticoid nasal drops for at least 6 weeks prior to the start of the study. There were no changes to the study methods after the trial commenced.

The following diagnostic procedures were performed during study enrolment.

1. A careful examination of the eyes, ears, nose, and throat was performed on all patients. The severity of perennial allergic rhinitis (AR) was assessed using the allergic rhinitis and its impact on asthma (ARIA) guidelines [11].
2. The skin prick test (SPT) was performed using inhalant allergens (HAL Allergy B.V., Leiden, Netherlands) from the following panel: *D. pteronyssinus, D. farinae*, 5 mixed grasses (*Phleum pratense*, *Dactylis glomerata*, *Anthoxanthum odoratum*, *Lolium perenne*, and *Poa pratensis*), mixed tree, mugwort, *Alternaria*, *Cladosporium*, and dog and cat allergens. Positive (10 mg/ml of histamine) and negative (saline) controls were also included. A house dust mite allergy was defined as a positive skin test for *D. pteronyssinus and D. farinae* allergens, with a minimum wheal diameter 3 mm greater than the negative control [12]. Patients with negative tests for histamine sensitivity were excluded from further analyses. The IgE measurement is described below.
3. Nasal provocation test (NPTs) were conducted using acoustic rhinometry with a commercial *D. pteronyssinus* allergen. The concentration was 10,000 AU/ml, and the mixture was delivered as 1 puff per nostril (HAL Allergy B.V., Leiden, Netherlands), using the method described by Bachert et al. [13] and Dordal et al. [14]. A reduction in the peak nasal inspiratory flow greater than or equal to 40% and an increase in symptoms greater than or equal to five points were considered to be positive NPT criteria, as defined by Bachert et al. [13] and Dordal et al. [14].

### Interventions

All participants were randomized to the active treatment and received PURETHAL Mites (20,000 AUeq/ml, HAL Allergy B.V., Leiden, Netherlands) or placebo. PURETHAL Mites containing major allergen equivalents of 14.0 μg/ml (group 1) and 20.0 μg/ml (group 2) were measured by ELISA in the extract prior to modification and adsorption on aluminium hydroxide). PURETHAL Mites were administered as perennial therapy using the following regimen: 1 dose (0.1 ml), 2 doses (0.2 ml), and 3 doses (0.5 ml) every week, and 0.5 ml every 4 weeks for 24 months. Using this schedule, the average cumulative dose was 560,500 BAU (bioequivalent allergy units) administered to each patient undergoing active treatment for the 2 years of the study.

The placebo was administered using the same protocol as the PURETHAL. The placebo was a sterile aluminium hydroxide suspension packed in a bottle similar to that of the active drug and packed in the same type of unidentified white boxes, with only the ID number of the patient and key number of the drug. All key codes used to identify the active drug or placebo were locked by an independent coordinator who did not participate in the study until the study was complete.

For blinding purposes, all patients received the same volume and same number of injections.

The rescue medication (oral antihistamines, nasal corticosteroids, oral corticosteroids) were provided to the participants, who were instructed to use them according to a stepwise regimen for the management of allergic rhinitis (see below).

### Outcomes

#### Assessment of efficacy

The symptoms and medication score were presented as the average adjusted symptom score (AAdSS). The primary endpoint was the change from baseline to end of the trial in the mean AAdSS and the TCRS difference in the least square means for the label compared to the placebo. The AAdSS was accepted for use as a primary end-point in rhinoconjunctivitis allergen immunotherapy trials [15]. This score includes the nasal and ocular total symptoms score associated with the house dust mite allergy as the rhinoconjunctivitis total symptoms score (RTSS), which can be adjusted for the use of symptomatic treatment [15]. Additionally, post hoc analysis was performed with the total combined rhinitis score (TCRS), which focused on nasal domain symptoms and the medication used for allergic rhinitis [15].

Patients recorded their nasal and ocular symptoms for the medication they used every day during the observation period (1 year before the trial and 2 years during the AIT). Four nasal symptoms (sneezing, rhinorrhea, pruritus and congestion) and two ocular symptoms (pruritus and tearing) were monitored. Each day, the patient rated the severity of each individual symptom over the past 24 h on a four-point scale: 0 = no symptoms, 1 = mild symptoms, 2 = moderate symptoms, and 3 = severe symptoms.

The rescue medication score was based on the WAO recommendations: 1 point for antihistamines, 2 points for nasal corticosteroids and 3 for oral corticosteroids [16].

The secondary outcome measurements included the quality of life, reduction of symptom score, safety assessment and monitoring of IgE to *Der p 1*, *Der p 2* and IgG_4_. The local reactions were assessed 30 min after injection and measured in cm. The systemic reactions were graded according to the EAACI criteria [17].

### Quality of life

Patient quality of life was evaluated with the rhinoconjunctivitis quality of life questionnaire (RQLQ) score for adults using questionnaires administered every year during the observation period [18]. Questionnaires were collected by medical staff.

### Allergen-specific IgE and IgG4

At baseline, after 1 year and at the end of the trial, serum-specific IgE, IgG and IgG4 levels to HDM (*D. pteronyssinus*, *D. farinae*) and to *Der p1* and *Der p2* were determined by Immuno CAP (ThermoFisher Scientific, Uppsala, Sweden), according to the manufacturer’s instructions.

The results were considered to be positive when the sIgE concentration was greater than 0.35 IU/ml. Additionally, the allergen-specific IgE and serum allergen-specific IgG4 response to *Der p1* and *Der p2* were measured using the same immunoenzymatic test. These markings were made at the start and end of the study.

All patient data were collected at baseline and after 1 and 2 years of treatment at the study centre.

### Sample size

The number of included patients was based on a power calculation that took into account the expected effect size, the standard deviation of the outcomes and the ordinal variable for the comparative study. The following formula was used to compare two proportions: N = 16p (1 − p)/(po − p1)^2^ and p = (p0 + p1)/2 for p0 = 0.2 and p1 = 0.15.

**Fig. 1:**
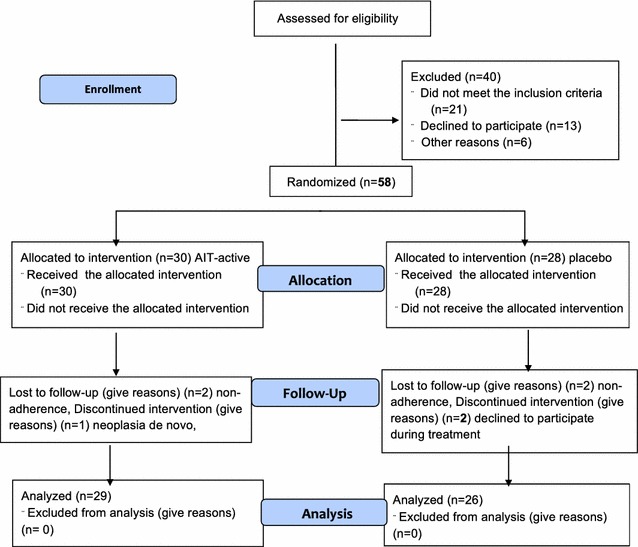
Number of participants assessed for eligibility who completed the study

Using a double-blind method, fifty-eight patients were individually randomised in comparable numbers to one of two parallel groups (Fig. 1).

### Randomization procedure

Using a computer—generated randomization list (block size of 6), eligible participants were randomized 1:1 to receive placebo or activate treatment with HDM extract. Sequentially numbered containers of PURETHAL or placebo were used to implement the random allocation sequence for all participants. The numbers were generated by a computer system and were under the control of the study coordinator. The investigators, subjects and personnel remained blinded throughout the study, until the database was locked.

### Statistical methods

The statistical analysis was performed using Statistica software, version 8.12 (SoftPol, Cracow, Poland). The Intent-to-treat (ITT) included all randomized patients. The modified ITT population included all randomised participants with an evaluable endpoint. The per-protocol patients (PP) included the participants who complied with the study treatment, which was defined as taking 80% or more of the study therapy for the duration of the study. The primary analysis based on the AAdSS difference from baseline using the ANOVA model. Post hoc analysis of TCRS was performed based on the same model. Secondary outcomes were assessed using appropriate non-parametric methods (Chi square, Wilcoxon test). Differences were considered to be significant for *p* < 0.05.

## Results

The participant numbers at enrolment, randomisation, treatment and follow-up are presented in Fig. 1. The baseline characteristics for each group are presented in Table 1.

**Table 1:** Patient characteristics at baseline

	Active n = 30	Placebo n = 28	*p* value
Age (years)	68.1 ± 5.9	69.2 ± 6.3	0.35
Male/female ratio	12/18	11/17	0.45
Duration of rhinitis (years)	4.4 ± 1.4	6.9 ± 2.1	0.23
Number of subjects with asthma	0	0	–
Number of patients with eczema	2	1	0.21
Number of smokers	6	5	0.67
Number of patients with stable coronary disease	8	10	0.17
Number of patients with arterial hypertension	12	9	0.22
Number of patients with diabetes	4	5	0.39
Mean weekly nasal symptom score	3.21 ± 0.93	3.11 ± 0.54	0.18
Mean weekly non-nasal symptom score	3.64 ± 0.55	3.21 ± 0.9	0.11
Mean weekly medication score	0.45 ± 0.17	0.51 ± 0.09	0.59
Total IgE	187.43 ± 64.01	201.9 ± 83.22	0.19
Specific IgE to Der p (kU/l)	24.9 ± 10.11	27.31 ± 13.9	0.4
Specific IgE to Der f (kU/l)	17.45 ± 8.31	15.9 ± 10.5	0.19

A total of 58 participants were enrolled in the study, and 55 (93%) completed the primary endpoint evaluation at 2 years (PP).

Twenty-nine subjects in the AIT group and twenty-six subjects in the placebo group completed the 2-year observation period.

Adherence to the injections was recorded by staff for the entire study. In the ITT population, 100% of the completed participants received > 75% of their injections, 93% of the participants received > 80% and 98 participants received > 90% throughout the 2-year treatment.

### Primary endpoints

After 24 months of AIT for HDM allergy, a significant clinical effect was observed based on the AAdSS compared to the baseline and placebo groups. In the ITT population, the AAdSS significantly decreased by approximately 64% in the active group: 4.27 ± 1.58 from baseline to 1.82 ± 0.71 after 2 years of AIT, *p* < 0.05. The active treatment group showed a 52% improvement after 24 months of AIT compared to the placebo group: 1.82 ± 0.71 versus 3.97 ± 0.96, *p* < 0.05. The results for the ITT and modified ITT populations are presented in Table 2.

**Table 2:** Efficacy of AIT during therapy compared to placebo

Patients	AAdSS ± SD		
	Baseline	After 2 years AIT	Difference in the adjusted means^a^
AIT active; n = 30	4.27 ± 1.58	1.82 ± 0.71	− 3.39
ITT population			
AIT placebo; n = 28	4.26 ± 1.6	3.97 ± 0.96	− 0.82
ITT population			
AIT active; n = 29	4.34 ± 1.71	1.93 ± 0.64	− 3.51
modified ITT population			
AIT placebo; n = 26	4.46 ± 1.69	3.92 ± 1.11	− 0.87
modified ITT population			

*AAdSS* average adjusted symptoms score

^a^The differences between AAdSS after 2 years of AIT and baseline

**Fig. 2:**
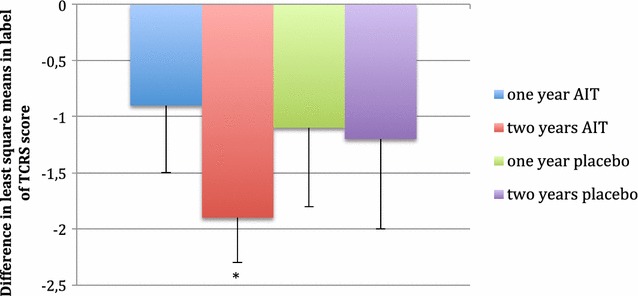
Decrease in the TCRS score in the active and placebo group during the study. Asterisk least square mean change in TCRS score was significant compared to baseline, *p* < 0.05

The post hoc analysis of TCRS showed that it was significantly decreased after 2 years of AIT in the ITT population (Fig. 2).

### Secondary outcomes

#### Immunological markers

**Fig. 3:**
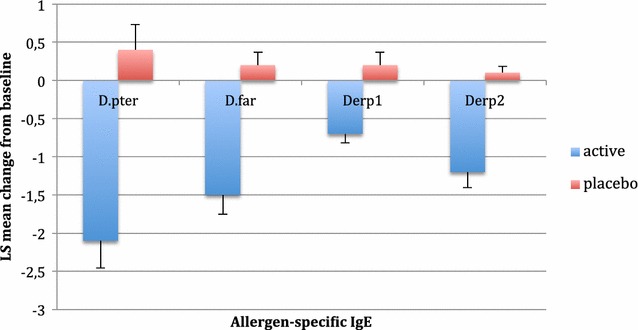
Decrease in HDM-specific IgE levels (SE) compared with placebo 3 and 6 years after the start of treatment. *D. pter*—the mean change of IgE against *D. pteronyssinus* from baseline after 2 years of AIT or placebo, *D. far*—the mean change of IgE against *D. farinae* from baseline after 2 years of AIT or placebo, Derp1—the mean change of IgE against antigen *D. pter* 1 from baseline after 2 years of AIT or placebo, *D. pter 2*—the mean change of IgE against antigen *D. pter* 2 from baseline after 2 years of AIT or placebo. There were significant changes between the active and placebo group in all analysed parameters

**Fig. 4:**
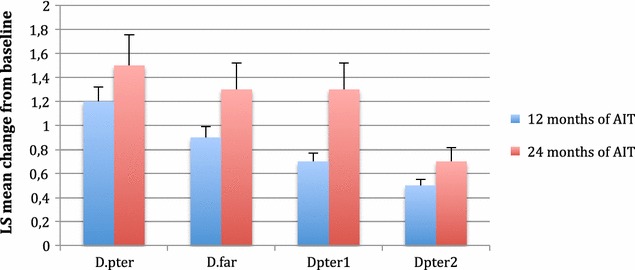
Increase in HDM-specific IgG4 levels (SE) after 12 and 24 months following the start of treatment. *D. pter_*—the mean change of IgG_4_ against *D. pteronyssinus* from baseline, *D. far*—mean change of IgG_4_ against *D. farinae* from baseline, *Derp1—the mean change of IgG*_*4*_ *against antigen D pter 1* from baseline, *Derp2*—the mean change of IgG_4_ against antigen *D pter 2* from baseline

Serum specific IgE against *D. pteronyssinus*, *D. farinae, D pter 1* and *D pter 2* decreased in the ITT population during the AIT trial (Fig. 3). Serum specific IgG4 against *D. pteronyssinus*, *D. farinae*, *Der p1*, and *Der p2* increased during the AIT trial in the study group (Fig. 4). The concentration of serum IgG4 in the placebo group was constant, with low levels of IgG4 against the analysed allergens.

#### Quality of life

In the ITT population, the quality of life (based on RQLQ) was significantly improved in patients who received AIT, from 1.82 (95% CI 1.54–1.92) to 1.26 (95% CI 1.09–1.55). In the placebo group, the quality of life was significantly lower, with a constant level of 1.74 (95% CI 1.27–1.86) during the trial.

#### Safety assessment

There were 2 mild systemic anaphylactic reactions (degree I) and no degree II, III or IV reactions in the active group during the AIT therapy. Erythema or wheals measuring < 5 cm was observed after 49 (4.11%) injections of PURETHAL. Wheals > 5 cm were observed after 19 (1.12%) were administered to the active treatment group There were no adverse reactions in the placebo group.

## Discussion

In this study, the AAdSS, as the primary endpoint of our analysis, decreased significantly during the AIT in the active group. This study is the first double-blind, placebo-controlled AIT trial performed in elderly patients with allergies to house dust mites. Immunotherapy in patients older than 65 years of age is uncommon. However, the prevalence of IgE-dependent allergic rhinitis and other atopic diseases in elderly patients is increasing [2, 19]. There is only one other DBPC study investigating sublingual immunotherapy for house dust mite allergies in similar elderly people [9]. The results of the current study are similar to that study (i.e., the same clinical effects after 3 years of AIT and comparable safety). These results are also comparable with other studies, including similar parameters in younger patients after AIT for house dust mites [20, 21].

There was also a significant improvement in the TCRS, based on the analysis of only the rhinitis domain as the primary endpoint of our study. This finding is particularly important for elderly patients because nasal problems significantly reduce patient quality of life [22].

The changes in the examined immune parameters correlated with the clinical improvement in the active group. The increase of IgG4 for *D. pteronyssinus, D. farinae,* Der p1 and Der p2 during and after AIT was conclusive. At the same time, the concentration of allergen-specific IgE for respective allergens was generally decreased but not in all patients. The influence of AIT on the concentration of IgG4 and specific IgE has been observed by other authors in younger allergic patients [23]. These results may be evidence that the immune system is able to create tolerance to allergens in the elderly. However, do not forget that immunosenescence is an important event during ageing. It also influences local nasal immune reactions. The ageing immune system incurs many changes, including a decrease in non-specific immune responses, with a decline in the activity of phagocytes and cytotoxic cells. Significant changes in the profiles of T lymphocytes during ageing have been observed [24]. The Th2 profile becomes predominant. The immune systems of elderly people respond poorly to new antigens. This response is caused by a predominance of memory lymphocytes and a significant reduction of native cells. However, it seems that these changes are not crucial in the incidence of allergies [25–27].

The PURETHAL used in the study is an allergoid of high efficacy and safety, regardless of the dosage regimen and type of allergen; this finding has been confirmed in several studies [28–30]. The obtained result suggests that we observed the same immunomodulatory effect that has been observed in young patients despite the ageing of the immune system. Additionally, reducing the need for symptom-targeted drugs and a primary reliance on antihistamines improved the safety profile of treatment for the elderly. In older patients, the most frequent adverse reactions are to antihistamine drugs [31]. This study supports the use of immunotherapy in elderly patients and demonstrates an acceptable safety profile without any clinically relevant systemic reactions during the 3 years of therapy. The observed improvement in quality of life is important in elderly patients and corroborated the efficacy of AIT in this age group [9, 10, 32].

The primary limitation of the study is the relatively small group of analysed patients. We also did not analyse specific IgE and IgG4 for other mite antigens, preventing the assessment of diverse mite allergies in the patients studied, as well as the different responses to AIT. Furthermore, we focused on typical allergic adverse reactions, and non-allergic types of adverse events, such as hot flushes, headache, nausea, diarrhoea, weakness, rise in body temperature, and nasal blockage were not analysed. Therefore, this observation may be incomplete for a total evaluation.

## Conclusion

This study showed that AIT to house dust mite allergens resulted in a significant clinical improvement in the active group compared to the placebo group. This therapy was well tolerated. These observations support the use of AIT in the elderly and indicate the need for larger studies.

## Abbreviations

AAdSS: average adjusted symptom score
AIT: allergen immunotherapy
HDM: house dust mites
NPT: nasal provocation test
nsIgE: nasal specific IgE
RQLQ: the rhinoconjunctivitis quality of life questionnaire
sIgE: allergen specific IgE
SPT: skin prick tests
SMS: symptoms medications score
